# Unsupervised learning‐derived phenotypes for personalized fluid management in critically ill patients with heart failure: A multicenter study

**DOI:** 10.1002/ctm2.70081

**Published:** 2024-11-08

**Authors:** Chengjian Guan, Angwei Gong, Yan Zhao, Hangtian Yu, Shuaidan Zhang, Zhiyi Xie, Yehui Jin, Xiuchun Yang, Jingchao Lu, Bing Xiao

**Affiliations:** ^1^ Department of Cardiology The Second Hospital of Hebei Medical University Shijiazhuang People's Republic of China

Dear Editor,

Fluid balance management in critically ill heart failure (HF) patients remains a formidable clinical challenge. While clinicians typically aim for net negative fluid balance to alleviate symptoms, recent studies employing fixed strategies have yielded inconsistent results.^1^, ^2^ The 2024 Heart Failure Association guidelines of the European Society of Cardiology emphasized the importance of individualized fluid balance strategies, particularly for critically ill patients.^3^ Our study introduces a novel approach using unsupervised learning to identify four distinct phenotypes of critically ill HF patients, each with unique clinical characteristics and fluid balance requirements. To facilitate clinical application, we have developed a user‐friendly interface that enables rapid phenotype identification and customized fluid management.

We utilized two non‐overlapping databases: III‐CareVue subset and IV versions of the Intensive Care Medical Information Marketplace (MIMIC)^4^ for training cohorts and the eICU Collaborative Research Database (eICU)^5^ for external validation (Method S1). The MIMIC cohort comprised 5998 patients, while the eICU cohort included 2549 patients (Figure S1). We initially extracted 56 variables from the first day of ICU admission. After eliminating variables with more than 30% missing data, 47 variables remained, encompassing demographics, comorbidities, laboratory values, vital signs, interventions, and severity scores. To ensure a balanced contribution of characteristics, all data underwent cleaning and normalization (Method S2, Figure S2). In‐hospital mortality served as our primary outcome, with ICU length of stay and total hospital length of stay as secondary outcomes.

Uniform Manifold Approximation and Projection (UMAP) was used to determine that there were no differences in clinical characteristics between the two training databases (Figure S3). To classify patients, we applied the K‐prototypes clustering algorithm, which effectively accommodates mixed numerical and categorical attributes while preserving the characteristics of factorial variables (Method S3). The optimal number of clusters was determined using standard tests, considering both statistical metrics and clinical relevance. This approach ultimately identified four distinct phenotypes (Figure S4).

Comparative analysis of these phenotypes revealed distinct clinical profiles (Figure 1, Table 1, Table S1). Phenotype A was characterized by aggressive interventions and inflammation, including high rates of vasoactive drug use, antibiotic use, and mechanical ventilation. This group also exhibited the highest white blood cell count and chloride levels, coupled with the lowest platelet count. Phenotype B represented the mildest form with the most favourable prognosis. Phenotype C was distinguished by the highest mean age, lowest body weight, higher comorbidity burden, and second‐highest mortality rate, despite having the lowest Sequential Organ Failure Assessment (SOFA) score. Phenotype D presented the most severe clinical profile with a poor prognosis. Short‐and long‐term survival outcomes differed significantly among these phenotypes, with Phenotype D showing the worst prognosis and Phenotype B the best prognosis (Figure 2A,B). To validate our findings, we conducted correlation analysis among continuous variables and excluded highly correlated factors before re‐clustering. The resulting phenotypes retained consistent characteristics, confirming the stability of our clustering method (Figures S5–S7).

**FIGURE 1:**
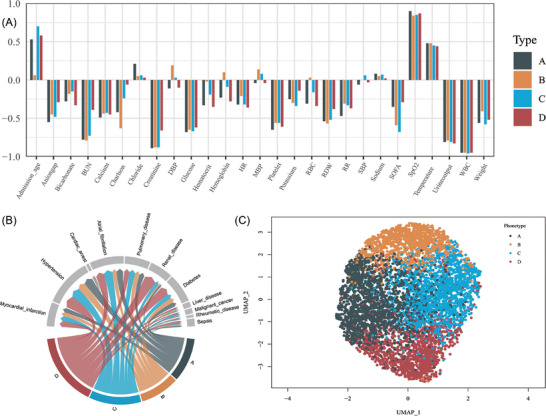
Characteristics of HF patients with different phenotypes. (A) Histogram of continuous variables for different phenotypes scaled to −1 to 1. (B) Chord diagram of the proportion of comorbidities by phenotype. Arc links between nodes with weights (width of arc). (C) Using Uniform Manifold Approximation and Projection (UMAP) to reduce dimensionality and visualize differences among the four phenotypes. BUN, blood urea nitrogen; DBP, diastolic blood pressure; HR, heart rate; MBP, mean blood pressure; RBC, red blood cell; RDW, red cell distribution width; RR, respiratory rate; SBP, systolic blood pressure; SOFA, Sequential Organ Failure Assessment; SpO_2_, percutaneous arterial oxygen saturation; WBC, white blood cell.

**TABLE 1:** Comparison of baseline characteristics of patients with four phenotypes.

	Heart failure phenotype				
Variables	A (*n* = 1949)	B (*n* = 1098)	C (*n *= 1677)	D (*n* = 1274)	*P‐value* ^1^
**Clinical information**					
Admission Age, M (Q₁, Q₃)	73.42 (65.64,79.90)	57.46 (49.14,63.53)	79.73 (74.05,84.17)	75.73 (68.40,81.90)	<.001***
Gender, *n* (%)					<.001***
Female	1077 (55.26)	340 (30.97)	765 (45.62)	417 (32.73)	
Male	872 (44.74)	758 (69.03)	912 (54.38)	857 (67.27)	
Race, *n* (%)					<.001***
White	1327 (68.09)	658 (59.93)	1200 (71.56)	880 (69.07)	
Black	114 (5.85)	135 (12.30)	126 (7.51)	125 (9.81)	
Other	508 (26.06)	305 (27.78)	351 (20.93)	269 (21.11)	
Weight, M (Q₁, Q₃)	79.50 (66.80,94.25)	94.78 (77.70,115.39)	76.80 (65.00,90.25)	83.18 (70.10,98.15)	<.001***
Admission type, *n* (%)					<.001***
Emergency	1398 (71.73)	918 (83.61)	1443 (86.05)	1043 (81.87)	
Floor	551 (28.27)	180 (16.39)	234 (13.95)	231 (18.13)	
First care unit, *n* (%)					<.001***
CICU	1370 (70.29)	647 (58.93)	836 (49.85)	661 (51.88)	
MICU	411 (21.09)	322 (29.33)	514 (30.65)	458 (35.95)	
SICU	83 (4.26)	64 (5.83)	189 (11.27)	91 (7.14)	
NSICU	5 (.26)	22 (2.00)	54 (3.22)	18 (1.41)	
TSICU	80 (4.10)	43 (3.92)	84 (5.01)	46 (3.61)	
**Comorbidities**					
Charlson comorbidity index, M (Q₁, Q₃)	6.00 (5.00,7.00)	4.00 (3.00,5.00)	7.00 (6.00,8.00)	8.00 (7.00,10.00)	<.001***
MI, *n* (%)					<.001***
No	1324 (67.93)	775 (70.58)	1070 (63.80)	755 (59.26)	
Yes	625 (32.07)	323 (29.42)	607 (36.20)	519 (40.74)	
Hypertension, *n* (%)					<.001***
No	719 (36.89)	466 (42.44)	478 (28.50)	215 (16.88)	
Yes	1230 (63.11)	632 (57.56)	1199 (71.50)	1059 (83.12)	
Cardiac arrest, *n* (%)					.015**
No	1876 (96.25)	1042 (94.90)	1625 (96.90)	1210 (94.98)	
Yes	73 (3.75)	56 (5.10)	52 (3.10)	64 (5.02)	
AF, *n* (%)					<.001***
No	1043 (53.51)	764 (69.58)	640 (38.16)	527 (41.37)	
Yes	906 (46.49)	334 (30.42)	1037 (61.84)	747 (58.63)	
Pulmonary disease, *n* (%)					<.001***
No	1279 (65.62)	730 (66.48)	974 (58.08)	800 (62.79)	
Yes	670 (34.38)	368 (33.52)	703 (41.92)	474 (37.21)	
Renal disease, *n* (%)					<.001***
No	1688 (86.61)	973 (88.62)	1257 (74.96)	225 (17.66)	
Yes	261 (13.39)	125 (11.38)	420 (25.04)	1049 (82.34)	
Diabetes, *n* (%)					<.001***
No	1324 (67.93)	728 (66.30)	1119 (66.73)	430 (33.75)	
Yes	625 (32.07)	370 (33.70)	558 (33.27)	844 (66.25)	
Liver disease, *n* (%)					<.001***
No	1830 (93.89)	996 (90.71)	1609 (95.95)	1126 (88.38)	
Yes	119 (6.11)	102 (9.29)	68 (4.05)	148 (11.62)	
Malignant cancer, *n* (%)					<.001***
No	1842 (94.51)	1057 (96.27)	1492 (88.97)	1142 (89.64)	
Yes	107 (5.49)	41 (3.73)	185 (11.03)	132 (10.36)	
Rheumatic disease, *n* (%)					.003***
No	1867 (95.79)	1075 (97.91)	1598 (95.29)	1229 (96.47)	
Yes	82 (4.21)	23 (2.09)	79 (4.71)	45 (3.53)	
Sepsis, *n* (%)					<.001***
No	1747 (89.64)	998 (90.89)	1564 (93.26)	1037 (81.40)	
Yes	202 (10.36)	100 (9.11)	113 (6.74)	237 (18.60)	
SOFA, M (Q₁, Q₃)	6.00 (4.00,8.00)	4.00 (2.00,6.00)	3.00 (2.00,4.00)	7.00 (5.00,9.00)	<.001***
**Interventions**					
Vasoactive drugs, *n* (%)					<.001***
No	461 (23.65)	755 (68.76)	1462 (87.18)	636 (49.92)	
Yes	1488 (76.35)	343 (31.24)	215 (12.82)	638 (50.08)	
Antibiotics, *n* (%)					<.001***
No	321 (16.47)	529 (48.18)	1025 (61.12)	415 (32.57)	
Yes	1628 (83.53)	569 (51.82)	652 (38.88)	859 (67.43)	
Diuretics, *n* (%)					.014**
No	935 (47.97)	467 (42.53)	734 (43.77)	575 (45.13)	
Yes	1014 (52.03)	631 (57.47)	943 (56.23)	699 (54.87)	
CRRT, *n* (%)					<.001***
No	1926 (98.82)	1087 (99.00)	1669 (99.52)	1114 (87.44)	
Yes	23 (1.18)	11 (1.00)	8 (.48)	160 (12.56)	
Mechanical ventilation, *n* (%)					<.001***
No	335 (17.19)	695 (63.30)	1351 (80.56)	796 (62.48)	
Yes	1614 (82.81)	403 (36.70)	326 (19.44)	478 (37.52)	
**Outcomes**					
Hospital death, *n* (%)					<.001***
No	1753 (89.94)	1024 (93.26)	1464 (87.30)	1024 (80.38)	
Yes	196 (10.06)	74 (6.74)	213 (12.70)	250 (19.62)	
Los_hospital, M (Q₁, Q₃)	10.70 (7.19,15.58)	9.41 (6.11,15.09)	8.96 (6.08,13.90)	11.26 (7.43,17.82)	<.001***
Los_icu, M (Q₁, Q₃)	4.09 (2.88,6.71)	3.95 (2.83,6.56)	3.68 (2.65,5.57)	4.09 (2.83,6.87)	<.001***

*Note*: M: Median, Q₁: 1st Quartile, Q₃: 3st Quartile, * < .05, ** < .01, *** < .001.

Abbreviations: AF, atrial fibrillation; CICU, cardiac intensive care unit; CRRT, continuous renal replacement therapy; Los, length of stay; MI, myocardial infarction; MICU, medical intensive care unit; NSICU, neurosurgical intensive care unit; SICU, surgical intensive care unit; SOFA, sequential organ failure assessment; TSICU, trauma surgical intensive care unit.

^1^ Continuous variable was tested using the Kruskal‐waills test. Chi‐square test was used for comparison between categorical variable.

**FIGURE 2:**
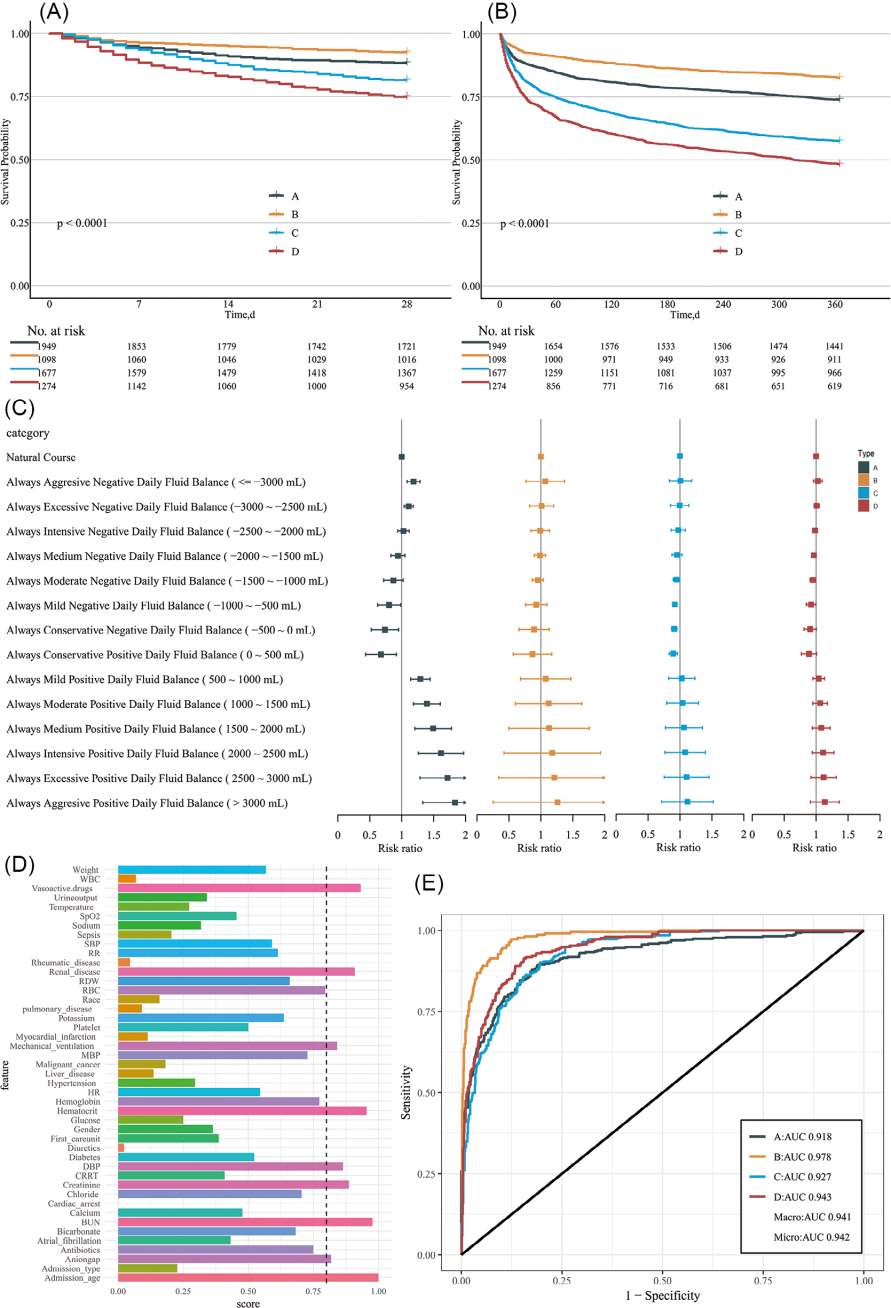
Phenotypic survival curves versus fluid equilibrium strategies, and performance of machine learning models Kaplan–Meier survival analyses at 28 days (A) and 1 year (B) for four phenotypes of HF. Association between daily fluid management strategies and in‐hospital mortality in four phenotypes (C). Feature selection (D) and ROC curve (E) for classifier learners. BUN, blood urea nitrogen; CRRT, continuous renal replacement therapy; DBP, diastolic blood pressure; HR, heart rate; JMIM, Joint Mutual Information Maximiza; MBP, mean blood pressure; RBC, red blood cell; RDW, red cell distribution width; ROC, receiver operating characteristic curve; RR, respiratory rate; SBP, systolic blood pressure; SpO_2_, percutaneous arterial oxygen saturation; WBC, white blood cell.

Additionally, we investigated phenotype‐specific fluid management strategies using net daily fluid balance data, adjusted for demographic factors, laboratory parameters, vital signs, and interventions. The impact of these strategies on in‐hospital mortality was analyzed using the parametric g‐formula (Method S4), focusing on the first seven days of ICU admission. Figure 2C illustrates how different fluid management strategies influence in‐hospital mortality across the four patient phenotypes over 7 days: Phenotype A presented with severe respiratory failure, shock, and inflammation, and benefited from fluid balances between −1000 and 500 mL daily. This aligned with recent studies on acute respiratory distress syndrome (ARDS) and ventilator‐related events, confirming the adverse effects of positive fluid balance on mechanical ventilation duration and mortality.^6^, ^7^ Phenotype C, despite milder clinical parameters, exhibited significantly higher mortality rates compared to Phenotype B, likely due to older age and multiple comorbidities. We recommended a daily net fluid balance ranging from −1500 and 500 mL for this group, underscoring the impact of age and frailty on HF prognosis.^8^ Phenotype D, characterized by severe metabolic derangements including acidosis, renal dysfunction, and sepsis, had the poorest prognosis. Our results suggested a more restrictive fluid strategy (−2000 to −500 mL daily), consistent with studies demonstrating adverse effects of positive fluid balance in populations with kidney disease and sepsis.^9^, ^10^ Phenotype B showed no clear benefit from specific fluid management strategies, warranting further investigation to determine whether this reflected the relative mildness of their condition or methodological limitations.

To facilitate the efficient classification of HF phenotypes across different cohorts, we developed a machine learning‐based classification model. The Joint Mutual Information Maximiza (JMIM) method identified nine variables with a feature importance score >.8, including age, blood urea nitrogen (BUN), hematocrit, vasoactive drugs, renal disease, creatinine, diastolic blood pressure (DBP), mechanical ventilation, and anion gap (Figure 2D). Based on benchmark tests (Table S3), we selected the Extreme Gradient Boosting (XGBoost) model for phenotype classification. The model achieved high predictive performance in MIMIC (AUC:.918–.943) (Figure 2E) and satisfactory performance in the eICU cohort used for external validation (AUC:.802–.907) (Figure S9). We evaluated the performance metrics and decision curve analysis of the model, and the results showed that the XGBoost model had good performance and clinical net benefit (Table S4, Figure S8). Then, we conducted an interpretability analysis to visualize the model's decision‐making process (Figures S10–S12). To support clinical application, we developed a web‐based tool (https://7kdtqk‐guanchengcheng.shinyapps.io/hf_phenotype/).

While our study provided valuable insights, several limitations warranted acknowledgement. First, the retrospective nature of the study precluded clear causal inference. Additionally, the absence of some important variables (such as ejection fraction and natriuretic peptides) might have led to the omission of potentially significant factors. Future randomized controlled trials were necessary to confirm the efficacy of phenotype‐specific fluid management strategies. Second, our analysis primarily focused on net fluid intake and its impact on prognosis. Further research was needed to explore the effects of infusion rates and individual fluid responsiveness on patient outcomes.

In summary, we have identified four distinct phenotypes of critically ill heart failure patients, each with unique clinical characteristics and fluid management needs. Our novel classification model and user interface facilitated rapid phenotype identification, enabling personalized fluid management strategies.

## AUTHOR CONTRIBUTIONS

Bing Xiao contributed to the research design. Chengjian Guan, Angwei Gong, and Yan Zhao contributed to data collection, data processing, and graphing. Chengjian Guan, Hangtian Yu, and Shuaidan Zhang conducted model construction and deployment. Zhiyi Xie, Yehui Jin, Xiuchun Yang, and Jingchao Lu contributed to data proofreading and formal analysis. Chengjian Guan and Angwei Gong contributed to the writing of the manuscript. Xiao Bing contributed to the review and editing. All authors have read and approved the final manuscript.

## CONFLICT OF INTEREST STATEMENT

The authors declare no conflict of interest.

## ETHICS STATEMENT

This study was conducted in accordance with the Declaration of Helsinki. Since the public databases used in this study all use de‐identified data, individual informed consent is not required, so we informed the Ethics Committee of this without a written report.

## Supporting information



Supporting information

## Data Availability

The data used in this study are from the MIMIC and the eICU databases. Although the database is de‐identified, the investigator still needs to obtain permission through the corresponding examination, so we have no right to disclose the data. The investigator may request data from the corresponding author of this article upon reasonable request after completing the examination and obtaining appropriate access.
