# Supplementary material for: Unsupervised learning‐derived phenotypes for personalized fluid management in critically ill patients with heart failure: A multicenter study
Source: Clin Transl Med. 2024 Nov 8;14(11):e70081. doi: 10.1002/ctm2.70081 (PMC11546239; doi:10.1002/ctm2.70081)
Supplement: Supplementary file 1 — Supporting information [file CTM2-14-e70081-s001.docx]

Supplementary materials

**Catalogue**

[Supplementary materials	1](#_Toc179283956)

[Supplementary methods	2](#_Toc179283957)

[Supplementary Method 1 MIMIC Database and eICU Database	2](#_Toc179283958)

[Supplementary Method 2 Data extraction and processing	2](#_Toc179283959)

[Supplementary Method 3 K-prototypes clustering algorithm	3](#_Toc179283960)

[Supplementary Method 4 Fitting Parameter g-formula	3](#_Toc179283961)

[Reference	4](#_Toc179283962)

[Supplementary tables	5](#_Toc179283963)

[Supplementary Table 1 Comparison of MIMIC Cohort Laboratory Parameters to Vital Signs Baseline	5](#_Toc179283964)

[Supplementary table 2 Risk ratio of fluid balance strategy on in-hospital mortality in HF patients with different phenotypes	7](#_Toc179283965)

[Supplementary Table 3 Accuracy of Model Benchmarking	8](#_Toc179283966)

[Supplementary Table 4 Evaluation Index of XGBoost Model	8](#_Toc179283967)

[Supplementary figures	9](#_Toc179283968)

[Supplementary figure 1 Flow chart of screening patients	9](#_Toc179283969)

[Supplementary figure 2 Missing proportion of each database	10](#_Toc179283970)

[Supplementary figure 3 MIMIC data distribution	11](#_Toc179283971)

[Supplementary figure 4 tot.withinss values for the number of clusters	12](#_Toc179283972)

[Supplementary Figure 5 Correlation of Continuous Variables in MIMIC Cohort	13](#_Toc179283973)

[Supplementary figure 6 Comparison of clinical features after re-clustering	14](#_Toc179283974)

[Supplementary figure 7 Kaplan-Meier curves of different phenotypes after re-clustering at 28 days and one year	15](#_Toc179283975)

[Supplementary figure 8 DCA curve for training validation	16](#_Toc179283976)

[Supplementary figure 9 ROC curve for external validation	17](#_Toc179283977)

[Supplementary figure 10 Importance of Model Features	18](#_Toc179283978)

[Supplementary figure 11 Dependency between variables and response variables	19](#_Toc179283979)

[Supplementary figure 12 Characteristic Contribution of Case	20](#_Toc179283980)

Supplementary methods

Supplementary Method 1 MIMIC Database and eICU Database

The Medical Information Mart for Intensive Care (MIMIC) database was created through a collaboration between the MIT Computational Physiology Laboratory, Beth Israel Deaconess Medical Center (BIDMC) at Harvard Medical School, and Philips Medical Corporation. Versions currently available include MIMIC-III and MIMIC-IV. MIMIC-IV^[1]^, updated to version 2.2, collected 431,231 hospital admissions and 73,181 ICU admissions from 299,712 patients from 2008 to 2019. MIMIC-III CareVue is a subset of the MIMIC-III database^[2]^, collecting admission records from 2001 to 2008, with no overlap with MIMIC-IV.

eICU Collaborative Research Database (eICU)^[3]^ is a large multi-center intensive care database provided by Philips Healthcare in collaboration with the MIT Computational Physiology Laboratory. The database contains more than 200,000 admissions from 2014 to 2015 for 139,367 patients admitted to 355 intensive care units at 208 hospitals in the United States.

Supplementary Method 2 Data extraction and processing

Structured Query Language (SQL) in PostgreSQL was used to extract data from two databases on patients admitted to the ICU during the first 24 hours. A total of 56 variables were included for analysis, including: (1) basic clinical information: sex, race, age, weight, height, admission type, first care unit; (2) comorbidities: myocardial infarction (MI), hypertension, cardiogenic shock, atrial fibrillation (AF), renal disease, pulmonary disease, liver disease, diabetes, malignant cancer, rheumatic disease, sepsis; (3) laboratory indicators: red blood cell (RBC), white blood cell (WBC), platelets, hemoglobin, hematocrit, red cell distribution width (RDW), anion gap, bicarbonate, chloride, blood urea nitrogen (BUN), creatinine, glucose, calcium, lactate, sodium, potassium, international normalized ratio (INR), prothrombin time (PT), partial thromboplastin time (PTT), potential of hydrogen (PH), B-type natriuretic peptide (BNP), C-reactive protein (CRP), magnesium (Mg), albumin and urine output; (4) Vital signs: heart rate (HR), respiratory rate (RR), systolic blood pressure (SBP), diastolic blood pressure (DBP), mean arterial pressure (MBP), temperature, partial pressure of carbon dioxide (PCO2), partial pressure of oxygen (PO2), total carbon dioxide (TotalCO2) and percutaneous arterial oxygen saturation (SpO2); (5) Interventions: Use of vasoactive drugs, antibiotics and diuretics, continuous renal replacement therapy (CRRT), mechanical ventilation; (6) Sequential Organ Failure Assessment (SOFA) score and Charlson comorbidity index on the first day of ICU admission. For variables with multiple daily measurements, the mean value was used for analysis. Variables with more than 30% missing values were removed, including INR, PT, PTT, PH, PCO2, PO2, TotalCO2, Height, BNP, CRP, Lactate, Mg and Albumin. Multivariate Imputation by Chained Equations (MICE) was used to interpolate the data and then normalization to balance the contribution of the features.

**Supplementary Method 3 K-prototypes clustering algorithm**

The K-Prototypes algorithm is a clustering technique designed to handle mixed data types, effectively clustering datasets that contain both numerical and categorical variables. It combines the principles of K-Means and K-Modes by calculating distances for numerical and categorical variables separately, allowing for comprehensive sample classification. For each type of variable, it employs different distance metrics: Euclidean distance for numerical variables and Hamming distance for categorical variables. This approach enhances the clustering results by considering the contributions of different variable types to the overall distance between samples. The algorithm begins with random selection of K initial prototypes, each consisting of values for both variable types. For each sample, the distances to the K prototypes are computed, assigning samples to the nearest cluster based on these distances. After samples are assigned, the prototypes are recalculated: the means for numerical variables and the modes for categorical variables. This process is repeated until the sample assignments no longer change, indicating convergence. K-Prototypes could effectively address the limitations of traditional clustering methods when dealing with mixed data types.

In clustering analysis, tot.withinss stands for "total within-cluster sum of squares." It measures the compactness of the clusters formed by quantifying how similar the members of each cluster are to one another. Within-cluster sum of squares (WSS) is calculated as the sum of the squared distances between each data point and the centroid of its respective cluster. The tot.withinss is the aggregate of these WSS values across all clusters, providing an overall indication of how well the data points are grouped. A lower tot.withinss suggests that the clusters are more compact and that the points within each cluster are closer together, indicating better clustering, while a higher value implies greater dispersion among the clusters.

Supplementary Method 4 Fitting Parameter g-formula

Cohort studies often involve longitudinal data with repeated measures, wherein each subject undergoes multiple assessments of the same throughout the study. In such research, addressing and controlling for time-dependent confounding presents a significant challenge. Time-dependent confounding variables evolve over time, and exposure to past or time-dependent confounding at a specific point can influence subsequent exposure. Conventional methods struggle to effectively control for time-dependent confounding and may introduce excessive adjustment bias or collision bias, leading to substantial errors in estimating exposure and outcome effects. The g-formula is an advanced method for analyzing longitudinal data to determine the effect of exposure on outcomes while controlling for time-dependent confounding factors common in repeated measures data. This approach employs Monte Carlo simulations based on counterfactual thinking^[4]^.

To investigate the impact of various fluid balance strategies on in-hospital mortality among patients with different heart failure phenotypes during the first seven days of ICU admission. We constructed the model using the parametric g-formula. This model incorporated common time-dependent confounding factors, including demographics (age, sex, race), laboratory variables (sodium, potassium, blood urea nitrogen, creatinine, glucose, anion gap, bicarbonate), vital signs (heart rate, respiratory rate, systolic blood pressure, diastolic blood pressure, mean blood pressure), and daily interventions (vasoactive drug dose, diuretic dose, albumin infusion, mechanical ventilation, and continuous renal replacement therapy).

Vasoactive drug doses follow the formula: NE = norepinephrine + epinephrine + phenylephrine/10 + dopamine/100 + metaraminol/8 + vasopressin*2.5 + angiotensin II*10^[5]^.

Diuretic equivalent doses follow the formula: PO furosemide ≈ 40 mg IV furosemide ≈ 40 mg PO or IV torsemide ≈ 1 mg PO or IV bumetanide ≈ 100 mg PO or IV ethacrynic acid diuretics^[6]^.

Following the methodology proposed by Liu et al.^[7]^, we categorized fluid balance into the 14 distinct strategies.

Reference

1.	Johnson, A.E.W., et al., *MIMIC-IV, a freely accessible electronic health record dataset.* Sci Data, 2023. **10**(1): p. 1.

2.	Johnson, A.E., et al., *MIMIC-III, a freely accessible critical care database.* Sci Data, 2016. **3**: p. 160035.

3.	Pollard, T.J., et al., *The eICU Collaborative Research Database, a freely available multi-center database for critical care research.* Sci Data, 2018. **5**: p. 180178.

4.	Keil, A.P., et al., *The parametric g-formula for time-to-event data: intuition and a worked example.* Epidemiology, 2014. **25**(6): p. 889-97.

5.	Goradia, S., et al., *Vasopressor dose equivalence: A scoping review and suggested formula.* J Crit Care, 2021. **61**: p. 233-240.

6.	Anisman, S.D., S.B. Erickson, and N.E. Morden, *How to prescribe loop diuretics in oedema.* Bmj, 2019. **364**: p. l359.

7.	Liu, P., et al., *Subphenotyping heterogeneous patients with chronic critical illness to guide individualised fluid balance treatment using machine learning: a retrospective cohort study.* EClinicalMedicine, 2023. **59**: p. 101970.

Supplementary tables

Supplementary Table 1 Comparison of MIMIC Cohort Laboratory Parameters to Vital Signs Baseline

| **Variables** | **Heart failure phenotype** | | | | ***P*** |
| --- | --- | --- | --- | --- | --- |
|  | **A (n = 1949)** | **B (n = 1098)** | **C (n = 1677)** | **D (n = 1274)** |  |
| **Laboratory parameters** | |  |  |  |  |
| WBC, M (Q₁, Q₃) | 12.50 (9.57,16.03) | 11.45 (8.59,15.05) | 10.30 (7.80,13.40) | 11.15 (8.15,15.14) | <.001 |
| RBC, M (Q₁, Q₃) | 3.31 (3.01,3.63) | 4.27 (3.85,4.69) | 3.74 (3.37,4.15) | 3.18 (2.86,3.57) | <.001 |
| Platelet, M (Q₁, Q₃) | 166.50 (126.33,217.50) | 212.00 (168.54,270.00) | 206.00 (159.67,269.00) | 185.33 (137.00,249.88) | <.001 |
| Hemoglobin, M (Q₁, Q₃) | 9.95 (9.05,10.95) | 12.70 (11.40,13.94) | 11.10 (9.90,12.30) | 9.40 (8.45,10.50) | <.001 |
| RDW, M (Q₁, Q₃) | 14.70 (13.80,15.87) | 14.25 (13.40,15.48) | 14.75 (13.80,16.15) | 16.08 (14.80,17.66) | <.001 |
| Hematocrit, M (Q₁, Q₃) | 29.63 (27.43,32.45) | 38.38 (34.40,41.90) | 33.60 (30.15,37.20) | 29.00 (26.30,32.19) | <.001 |
| Anion gap, M (Q₁, Q₃) | 13.00 (11.00,15.00) | 14.50 (12.67,16.58) | 14.25 (12.50,16.00) | 17.00 (14.67,19.80) | <.001 |
| Bicarbonate, M (Q₁, Q₃) | 23.00 (21.00,25.00) | 24.67 (22.00,28.00) | 25.50 (23.00,28.33) | 22.00 (19.50,25.00) | <.001 |
| BUN, M (Q₁, Q₃) | 20.50 (15.33,29.00) | 19.50 (14.50,28.00) | 25.00 (18.00,35.00) | 56.00 (39.00,80.00) | <.001 |
| Calcium, M (Q₁, Q₃) | 8.20 (7.80,8.65) | 8.60 (8.15,8.93) | 8.60 (8.20,8.90) | 8.47 (8.00,8.95) | <.001 |
| Chloride, M (Q₁, Q₃) | 106.50 (103.33,109.33) | 101.80 (98.00,105.00) | 102.00 (99.00,105.25) | 101.00 (96.67,105.50) | <.001 |
| Creatinine, M (Q₁, Q₃) | 1.00 (0.80,1.32) | 1.00 (0.80,1.32) | 1.10 (0.85,1.45) | 2.60 (1.83,4.05) | <.001 |
| Glucose, M (Q₁, Q₃) | 127.50 (107.00,155.33) | 134.00 (113.00,171.50) | 131.00 (110.50,161.33) | 144.50 (115.00,190.00) | <.001 |
| Sodium, M (Q₁, Q₃) | 138.67 (136.50,140.75) | 138.00 (135.76,140.50) | 139.00 (136.25,141.50) | 137.50 (134.06,140.31) | <.001 |
| Potassium, M (Q₁, Q₃) | 4.28 (3.97,4.60) | 4.13 (3.85,4.50) | 4.07 (3.80,4.40) | 4.54 (4.10,4.95) | <.001 |
| Urine output, M (Q₁, Q₃) | 1597.00 (1055.00,2395.00) | 2181.00 (1346.00,3330.00) | 1755.00 (1096.00,2680.00) | 1144.00 (495.00,2126.75) | <.001 |
| Vital signs | |  |  |  |  |
| HR, M (Q₁, Q₃) | 83.42 (76.66,91.57) | 90.12 (79.29,102.93) | 82.00 (71.96,94.82) | 80.04 (71.06,90.55) | <.001 |
| SBP, M (Q₁, Q₃) | 109.96 (103.36,117.24) | 113.08 (103.57,125.34) | 119.90 (108.58,133.44) | 111.22 (101.13,122.51) | <.001 |
| DBP, M (Q₁, Q₃) | 54.91 (50.02,59.66) | 67.51 (61.24,75.58) | 61.11 (54.50,67.75) | 55.24 (49.08,61.50) | <.001 |
| MBP, M (Q₁, Q₃) | 72.63 (67.98,76.98) | 81.11 (74.71,89.48) | 77.97 (72.04,86.16) | 71.58 (65.92,77.66) | <.001 |
| RR, M (Q₁, Q₃) | 17.73 (15.86,19.92) | 20.18 (17.76,23.01) | 20.00 (17.79,22.96) | 19.34 (17.15,21.85) | <.001 |
| Temperature, M (Q₁, Q₃) | 36.86 (36.55,37.22) | 36.83 (36.56,37.19) | 36.74 (36.49,36.99) | 36.67 (36.38,36.96) | <.001 |
| SpO2, M (Q₁, Q₃) | 97.97 (96.85,98.96) | 96.14 (94.79,97.56) | 96.50 (95.05,97.73) | 97.21 (95.76,98.47) | <.001 |
| #: Kruskal-waills test, χ²: Chi-square test | | | | | |
| M: Median, Q₁: 1st Quartile, Q₃: 3st Quartile | | | | | |
| WBC: white blood cell; RBC: red blood cell; RDW: red cell distribution width; BUN: blood urea nitrogen; HR: heart rate; RR: respiratory rate; SBP: systolic blood pressure; DBP: diastolic blood pressure; MBP: mean blood pressure; SpO2: percutaneous arterial oxygen saturation. | | | | | |

Supplementary table 2 Risk ratio of fluid balance strategy on in-hospital mortality in HF patients with different phenotypes

| **Category** | **A** | | **B** | | C | | D | |
| --- | --- | --- | --- | --- | --- | --- | --- | --- |
|  | **Risk ratio** | **95% CI** | **Risk ratio** | **95% CI** | **Risk ratio** | **95% CI** | **Risk ratio** | **95% CI** |
| Natural Course | 1 |  | 1 |  | 1 |  | 1 |  |
| Always Aggressive Negative Daily Fluid Balance (<= -3000 mL) | 1.186 | (1.084, 1.287) | 1.069 | (0.769, 1.369) | 1.007 | (0.832, 1.182) | 1.027 | (0.959, 1.094) |
| Always Excessive Negative Daily Fluid Balance (-3000 ~ -2500 mL) | 1.108 | (1.037, 1.180) | 1.012 | (0.822, 1.201) | 0.994 | (0.853, 1.136) | 1.008 | (0.968, 1.049) |
| Always Intensive Negative Daily Fluid Balance ( -2500 ~ -2000 mL) | 1.028 | (0.938, 1.117) | 0.990 | (0.844, 1.136) | 0.973 | (0.863, 1.083) | 0.983 | (0.963, 1.003) |
| Always Medium Negative Daily Fluid Balance ( -2000 ~ -1500 mL) | 0.941 | (0.831, 1.050) | 0.986 | (0.896, 1.075) | 0.953 | (0.874, 1.032) | **0.966** | **(0.941, 0.990)** |
| Always Moderate Negative Daily Fluid Balance ( -1500 ~ -1000 mL) | 0.869 | (0.716, 1.022) | 0.952 | (0.865, 1.039) | **0.945** | **(0.893, 0.998)** | **0.948** | **(0.904, 0.993)** |
| Always Mild Negative Daily Fluid Balance ( -1000 ~ -500 mL) | **0.802** | **(0.622, 0.982)** | 0.927 | (0.758, 1.096) | **0.918** | **(0.883, 0.953)** | **0.922** | **(0.846, 0.997)** |
| Always Conservative Negative Daily Fluid Balance ( -500 ~ 0 mL) | **0.737** | **(0.524, 0.950)** | 0.894 | (0.657, 1.130) | **0.910** | **(0.868, 0.952)** | 0.906 | (0.809, 1.002) |
| Always Conservative Positive Daily Fluid Balance (0 ~ 500 mL) | **0.675** | **(0.434, 0.917)** | 0.869 | (0.567, 1.172) | **0.895** | **(0.829, 0.960)** | 0.888 | (0.768, 1.007) |
| Always Mild Positive Daily Fluid Balance (500 ~ 1000 mL) | 1.292 | (1.140, 1.443) | 1.075 | (0.682, 1.469) | 1.028 | (0.822, 1.234) | 1.040 | (0.949, 1.130) |
| Always Moderate Positive Daily Fluid Balance (1000 ~ 1500 mL) | 1.394 | (1.183, 1.604) | 1.122 | (0.602, 1.641) | 1.040 | (0.790, 1.290) | 1.063 | (0.949, 1.177) |
| Always Medium Positive Daily Fluid Balance (1500 ~ 2000 mL) | 1.492 | (1.205, 1.779) | 1.128 | (0.499, 1.757) | 1.061 | (0.771, 1.351) | 1.080 | (0.941, 1.218) |
| Always Intensive Positive Daily Fluid Balance (2000 ~ 2500 mL) | 1.615 | (1.260, 1.970) | 1.178 | (0.422, 1.934) | 1.080 | (0.765, 1.396) | 1.109 | (0.939, 1.278) |
| Always Excessive Positive Daily Fluid Balance (2500 ~ 3000 mL) | 1.718 | (1.286, 2.150) | 1.211 | (0.340, 2.081) | 1.103 | (0.752, 1.454) | 1.117 | (0.919, 1.315) |
| Always Aggressive Positive Daily Fluid Balance (> 3000 mL) | 1.835 | (1.329, 2.340) | 1.261 | (0.253, 2.270) | 1.117 | (0.714, 1.519) | 1.135 | (0.909, 1.361) |

Supplementary Table 3 Accuracy of Model Benchmarking

| Model | Accuracy |
| --- | --- |
| XGBoost | 0.822269 |
|  |  |
| SVM | 0.795093 |
|  |  |
| Ranger | 0.817103 |
|  |  |
| ANN | 0.512042 |
|  |  |
| Naïve bayes | 0.792094 |
|  |  |
| KNN | 0.786925 |
|  |  |
| glmnet | 0.819274 |

XGBoost: eXtreme Gradient Boosting; SVM: Support Vector Machine; ANN: Artificial Neural Network; KNN: k-Nearest-Neighbor; glmnet: GLM with Elastic Net Regularization

Supplementary Table 4 Evaluation Index of XGBoost Model

|  | Sensitivity | Specificity | Precision | Recall | F1 Score | Kappa |
| --- | --- | --- | --- | --- | --- | --- |
| A | 0.7744 | 0.9033 | 0.7744 | 0.7744 | 0.7744 | 0.6777 |
| B | 0.8182 | 0.963 | 0.8182 | 0.8182 | 0.8182 | 0.7784 |
| C | 0.7612 | 0.9171 | 0.7612 | 0.7612 | 0.7612 | 0.6907 |
| D | 0.7373 | 0.9359 | 0.7373 | 0.7373 | 0.7373 | 0.7411 |
| Overall | 0.7728 | 0.9298 | 0.7728 | 0.7728 | 0.7728 | 0.6252 |

Supplementary figures

Supplementary figure 1 Flow chart of screening patients

MIMIC: Medical Information Mart for Intensive Care; eICU: eICU Collaborative Research Database.

Supplementary figure 2 Missing proportion of each database

Variables with more than 30% missing values were removed, including INR, PT, PTT, PH, PCO_2_, PO_2_, TotalCO_2_, Height, BNP, CRP, Lactate, Mg and Albumin.

Sofa: Sequential Organ Failure Assessment; BUN: blood urea nitrogen; SpO_2_: percutaneous arterial oxygen saturation; MBP: mean blood pressure; Resp_rate: respiratory rate; DBP: diastolic blood pressure; SBP: systolic blood pressure; RBC: red blood cell; WBC: white blood cell;

RDW: red cell distribution width; PT: prothrombin time; PTT: activated partial thromboplastin time; INR: international normalized ratio; PCO_2_: Partial Pressure of Carbon Dioxide; TotalCO_2_: Total Carbon Dioxide; PO_2_: Partial pressure of oxygen; BNP: Brain Natriuretic Peptide; CRP: C-reactive protein.

Supplementary figure 3 MIMIC data distribution

Supplementary figure 4 tot.withinss values for the number of clusters

The total within sum of squares indicates the differences within the cluster. It decreases as clusters increases.

Supplementary Figure 5 Correlation of Continuous Variables in MIMIC Cohort

Variables with correlation greater than 0.7: red blood cells, hematocrit, hemoglobin, systolic blood pressure, diastolic blood pressure, mean blood pressure.

Sofa: Sequential Organ Failure Assessment; BUN: blood urea nitrogen; SpO_2_: percutaneous arterial oxygen saturation; MBP: mean blood pressure; Resp_rate: respiratory rate; DBP: diastolic blood pressure; SBP: systolic blood pressure; RBC: red blood cell; WBC: white blood cell; RDW: red cell distribution width.

Supplementary figure 6 Comparison of clinical features after re-clustering

BUN: blood urea nitrogen; HR: heart rate; RDW: red cell distribution width; RR: respiratory rate; SOFA: Sequential Organ Failure Assessment; SpO_2_: percutaneous arterial oxygen saturation; WBC: white blood cell.

Supplementary figure 7 Kaplan-Meier curves of different phenotypes after re-clustering at 28 days and one year

Survival curves for the four phenotypes at 28 days (a) and one year (b) after re-clustering.

Supplementary figure 8 DCA curve for training validation

DCA: decision curve analysis.

Supplementary figure 9 ROC curve for external validation

ROC: receiver operating characteristic curve.

Supplementary figure 10 Importance of Model Features

BUN: blood urea nitrogen; DBP: diastolic blood pressure.

Supplementary figure 11 Dependency between variables and response variables

Vasopressor: vasoactive drugs; BUN: blood urea nitrogen; DBP: diastolic blood pressure.

Supplementary figure 12 Characteristic Contribution of Case

Figure S11 shows how the model predicts the heart failure phenotype, where green represents the positive effect, red represents the negative effect, and blue represents the total probability. In this figure, the patient is predicted to be phenotype C.

Vasopressor: vasoactive drugs; BUN: blood urea nitrogen; DBP: diastolic blood pressure.
